# Identification of Potential Biomarkers and Small Molecule Drugs for Cutaneous Melanoma Using Integrated Bioinformatic Analysis

**DOI:** 10.3389/fcell.2022.858633

**Published:** 2022-03-30

**Authors:** Yong Liu, Jiayi Sun, Dongran Han, Shengnan Cui, Xiaoning Yan

**Affiliations:** ^1^ School of Life Science, Beijing University of Chinese Medicine, Beijing, China; ^2^ Department of Dermatology, Shaanxi Provincial Hospital of Traditional Chinese Medicine, Xi’an, China; ^3^ Xiyuan Hospital, China Academy of Chinese Medical Sciences, Beijing, China; ^4^ Graduate School, China Academy of Chinese Medical Sciences, Beijing, China

**Keywords:** cutaneous melanoma, normal skin, prognosis, furazolidone, bioinformatics

## Abstract

**Background:** Cutaneous melanoma (CM) is a type of skin cancer with a high fatality rate, and its pathogenesis has not yet been fully elucidated.

**Methods:** We obtained the gene expression datasets of CM through the Gene Expression Omnibus (GEO) database. Subsequently, robust rank aggregation (RRA) method was used to identify differentially expressed genes (DEGs) between CM cases and normal skin controls. Gene functional annotation was performed to explore the potential function of the DEGs. We built the protein–protein interaction (PPI) network by the Interactive Gene database retrieval tool (STRING) and selected hub modules by Molecular Complexity Detection (MCODE). We furthered and validated our results using the TCGA-GTEX dataset. Finally, potential small molecule drugs were predicted by CMap database and verified by molecular docking method.

**Results:** A total of 135 DEGs were obtained by RRA synthesis analysis. GMPR, EMP3, SLC45A2, PDZD2, NPY1R, DLG5 and ADH1B were screened as potential targets for CM. Furazolidone was screened as a potential small molecule drug for the treatment of CM, and its mechanism may be related to the inhibition of CM cell proliferation by acting on GMPR.

**Conclusion:** We identified seven prognostic therapeutic targets associated with CM and furazolidone could be used as a potential drug for CM treatment, providing new prognostic markers, potential therapeutic targets and small molecule drugs for the treatment and prevention of CM.

## Introduction

Global statistics in 2020 showed that CM accounted for 1.7% of global cancer. Melanoma is the fifth most common cancer diagnosis in the United States by 2021, accounting for 5.6% of all cancer diagnoses. (Saginala et al., 2021). It is widely known, genetic changes facilitate early diagnosis and individualized treatment in patients with melanoma. In the past few years, microarray technology has been widely used in the analysis of gene expression profiles in the skin tissues of melanoma patients or experimental animals. Identifying gene-specific expression patterns makes it possible for people to discover the key gene changes in melanoma tissues and cells, which helps to understand the pathogenic mechanism of the disease or evaluate the treatment. However, there are some inconsistencies in these microarray studies, such as different laboratory conditions, racial differences in clinical samples, and differences in chip platforms. Therefore, it is of great significance to find an effective method to evaluate the results of different gene expression profiles.

**FIGURE 1:**
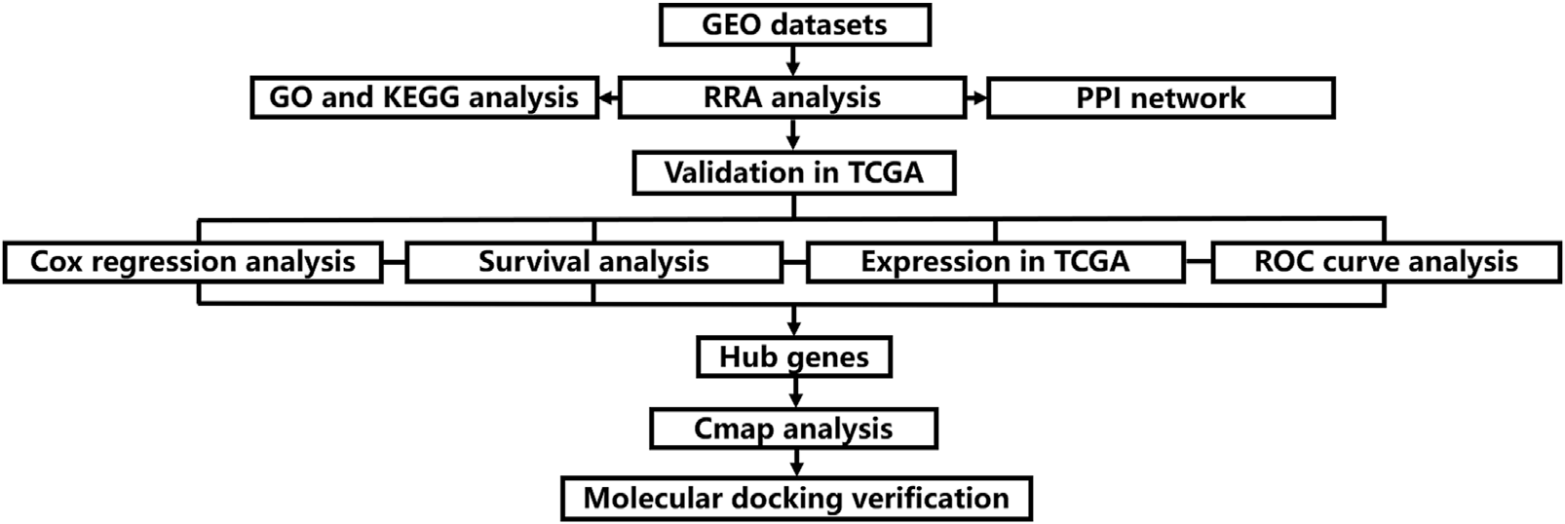
Flow chart of the present study.

RRA is a method that uses probability models to integrate ranked lists which has four key features: strong robustness to noise, ability to deal with incomplete ranking, giving significant scores to each element in the result ranking, and high computational efficiency (Kolde et al., 2012). Some studies have used it to integrate multiple sets of gene chip data lists, and achieved good results (Jia and Zhai, 2019). To our knowledge, previous CM studies did not use the RRA method to identify differentially expressed genes (DEGs), which facilitated this study. Therefore, we adopted a comprehensive bioinformatics approach to conduct a meta-analysis of gene expression between CM tissue and normal skin controls. In addition, according to the results of this analysis, gene enrichment and pathway annotation analysis were also carried out. In addition, based on the results of this analysis, the prediction of small molecule drugs to treat CM was also carried out, and the molecular docking method was used to verify and study the possible mechanism of action. The flow chart of this research is displayed in Figure 1.

## Materials and Methods

### Microarray Datasets of Cutaneous melanoma

The expression profiles of CM were retrieved from the Gene Expression Omnibus (GEO) database (https://www.ncbi.nlm.nih.gov/). The search strategy [“Cutaneous melanoma” (MeSH Terms) OR Cutaneous melanoma (All Fields)] and [“*Homo sapiens*” (Organism) and “Expression profiling by array” (Filter)] was adopted.

### Datasets Analyses

The expression microarray datasets were all standardized by quantiles. A linear model was used to assess differential expression between CM cases and normal skin controls using R package named “limma.” The |log2 fold change (FC)| > 1 and *p*-value < 0.05 were regarded as the cut-off criteria to determine DEGs (Liu et al., 2021).

### Robust Rank Aggregation Analysis

We integrated all the obtained up-regulated and down-regulated gene lists of each dataset using the “Robust Rank Aggregation” R package. Genes with *p*-value <0.05 and the fold change >1 were considered as significant genes. The adjusted *p*-value in the RRA tool indicate the possibility of ranking high of each gene in the final gene list.

### Functional and Pathway Enrichment Analysis

Gene ontology (GO) and Kyoto Encyclopedia of Genes and Genomes (KEGG) pathway analysis of the significant genes in RRA analysis were performed via The Database for Annotation, Visualization, and Integrated Discovery (DAVID 6.8, https://david.ncifcrf.gov/). *p*-value <0.05 was regarded as the cut-off criteria.

### Protein–Protein Interaction Network Establishment and Module Analysis

We uploaded the genes screened by the RRA method to the STRING database to obtain the interaction relationship information between genes, and the cutoff value was set to 0.4 to explore the interaction relationship between genes. Then, the interaction information was imported into Cytoscape, and a protein–protein interaction (PPI) network diagram was constructed, and sorted according to the degree value (Shannon et al., 2003). Modular analysis using Molecular Complex Detection (MCODE) plugin in Cytoscape with threshold nodes numbers >3, k-score = 2 and MCODE scores >3 (Bader and Hogue, 2003).

### Key Genes Validation Study

We use the CM RNA-seq dataset (461 CM cases) in the cancer genome atlas (TCGA) TCGA (https://portal.gdc.cancer.gov/) and 558 normal skin tissues RNA-seq dataset in GTEx (https://xenabrowser.net/datapages/) to verify the expression level of the selected key gene. Genes with *p*-value <0.05 were considered to be significant.

We utilized the CM dataset in TCGA to verify whether the DEGs analyzed by RRA had clinical prognostic significance. Through univariate Cox regression analysis, the Hazard Ratio (HR) value was calculated to judge the clinical prognostic significance of the variable, and the “Survminer” package was used to obtain the median value of the risk score, and the median value was used as the cut-off point to divide CM patients into high-risk groups and low-risk group, and Kaplan-Meier curve analysis was used to compare the survival time of the low-risk and high-risk groups. In addition, survival score and survival status curves and heatmaps were used to illustrate the distribution of CM patients in the two groups (high-risk and low-risk). We also performed ROC curve analysis to assess the predictive value of the results (the size of the area under the ROC curve was used to describe the predictive value). The above analysis was considered statistically significant at *p* < 0.05.

### Identification of Candidate Small Molecules

CMap is a program for predicting potential drugs that may induce biological states encoded by specific gene expression signatures (Lamb, 2007). We divided the finally screened differential genes into up-regulated and down-regulated groups, and imported them into CMap database, in order to explore small molecule drugs that might treat CM. A negative mean score indicates that the drug reverses the desired biological property and has potential therapeutic value (*p* < 0.05 for statistical significance).

### Molecular Docking Verification

We performed molecular docking verification between the small molecule drugs predicted in CMap and the potential target proteins of CM, and judged the reliability of drug treatment of CM by the size of the binding energy. The mol2 file format structures of the compounds were obtained from the PubChem database, and the crystal structures of the core targets were collected from the RCSB Protein Data Bank (PDB, http://www.rcsb.org/). First, target proteins were dehydrated and ligand-removed using PyMOL 2.3.2 software and stored in PDB format. The processed target protein was then imported into AutoDock Tools 1.5.6 software for hydrogenation, charge calculation and stored in PDBQT format. Mol2 files of small molecule drugs were imported into AutoDock Tools 1.5.6 software, total charge was detected, charge was assigned, flexible rotatable bonds were viewed and saved in PDBQT format. Grid box data for the protein of interest was obtained. Finally, run Autodock Vina 1.1.2 for molecular docking. Molecular docking results were visualized using PyMOL 2.3.2 software (Gaudreault et al., 2015).

## Results

### Identification of Differentially Expressed Genes in Cutaneous Melanoma

**FIGURE 2:**
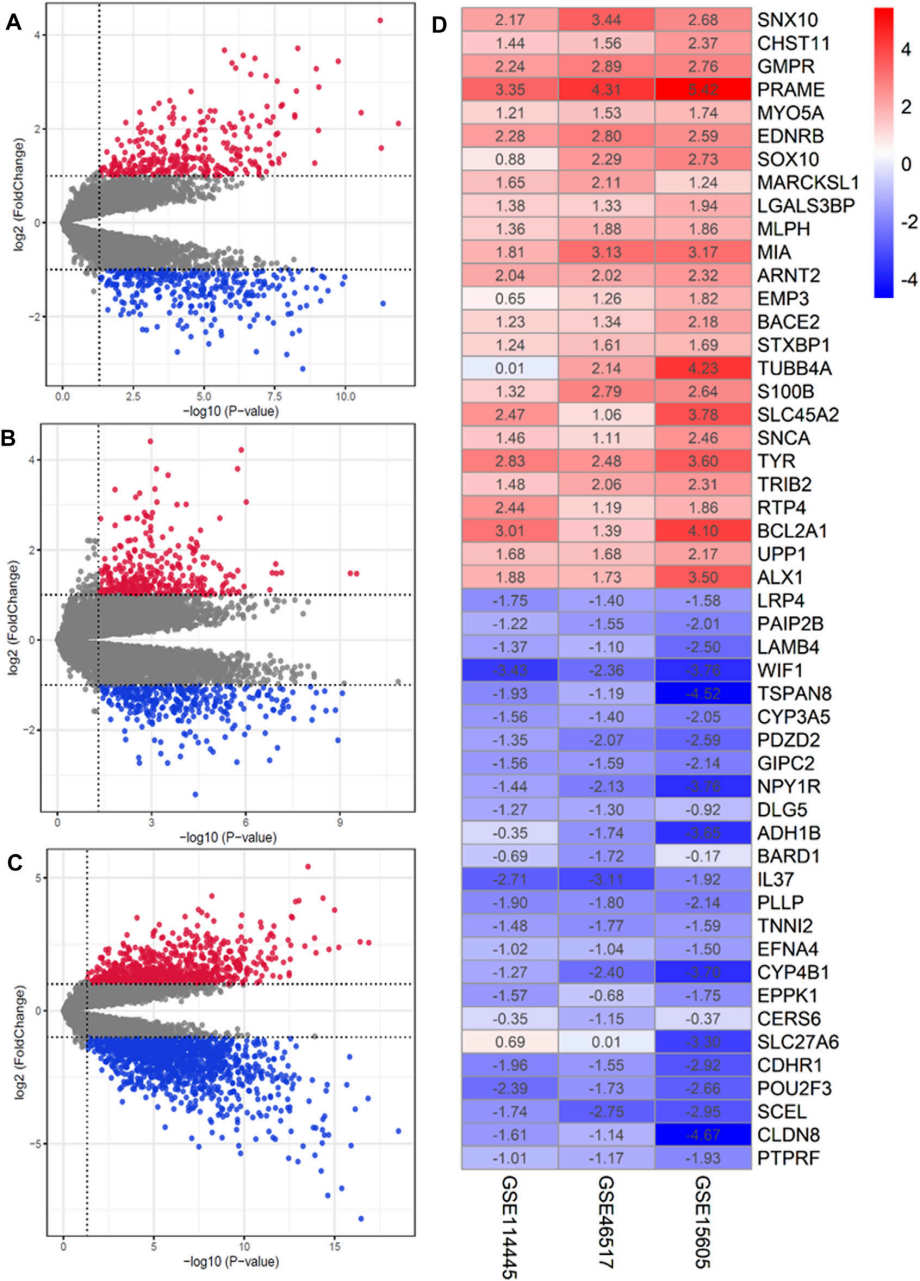
Identification of DEGs in GEO database. **(A–C)** Volcano plot of the DEGs in GSE15605, GSE46517 and GSE114445. Red and green indicate upregulated and downregulated genes (*p* < 0.05, LogFC ≥1 or ≤ −1), respectively. **(D)** Heatmap of the top 25 up- and down-regulated genes in the RRA analysis. The abscissa is the geo ID, and the ordinate is the gene name. Red represents logFC>0, blue represents logFC<0, and the values in the box represent the logFC values.

In accordance with our search strategy, we downloaded and analyzed three microarray datasets from the Gene Expression Omnibus (GEO) database, including GSE46517, GSE114445, and GSE15605. GSE46517 was platform-based on GPL96 and contained a total of 39 samples, including 31 primary melanoma samples and 8 normal skin samples. GSE114445 was based on the GPL570 platform, and a total of 22 samples were collected, including 16 primary melanoma sample and six normal skin samples. GSE15605 was based on the GPL570 platform and included a total of 62 samples, including 46 primary melanoma samples and 16 normal skin samples. Primary melanoma sample and normal skin sample from the three datasets were included in this study. The results are shown in Supplementary Table S1. The volcano plots of the three microarrays are shown in Figures 2A–C. A total of 135 DEGs (70 up-regulated and 65 down-regulated) were obtained through RRA integrated Analysis (Supplementary Table S1). The heatmap of the top 25 up and down-regulated genes is shown in Figure 2D.

### Functional and Network Analysis of Differentially Expressed Genes

GO functional enrichment analysis outcomes revealed that the most significant enrichment was positive regulation of cell proliferation, extracellular region and protein binding respectively among biological process (BP), cellular component (CC) and molecular function (MF). KEGG pathway enrichment analysis showed that pathways in cancer and transcriptional misregulation in cancer were significantly enriched, as shown in Supplementary Table S1.

**FIGURE 3:**
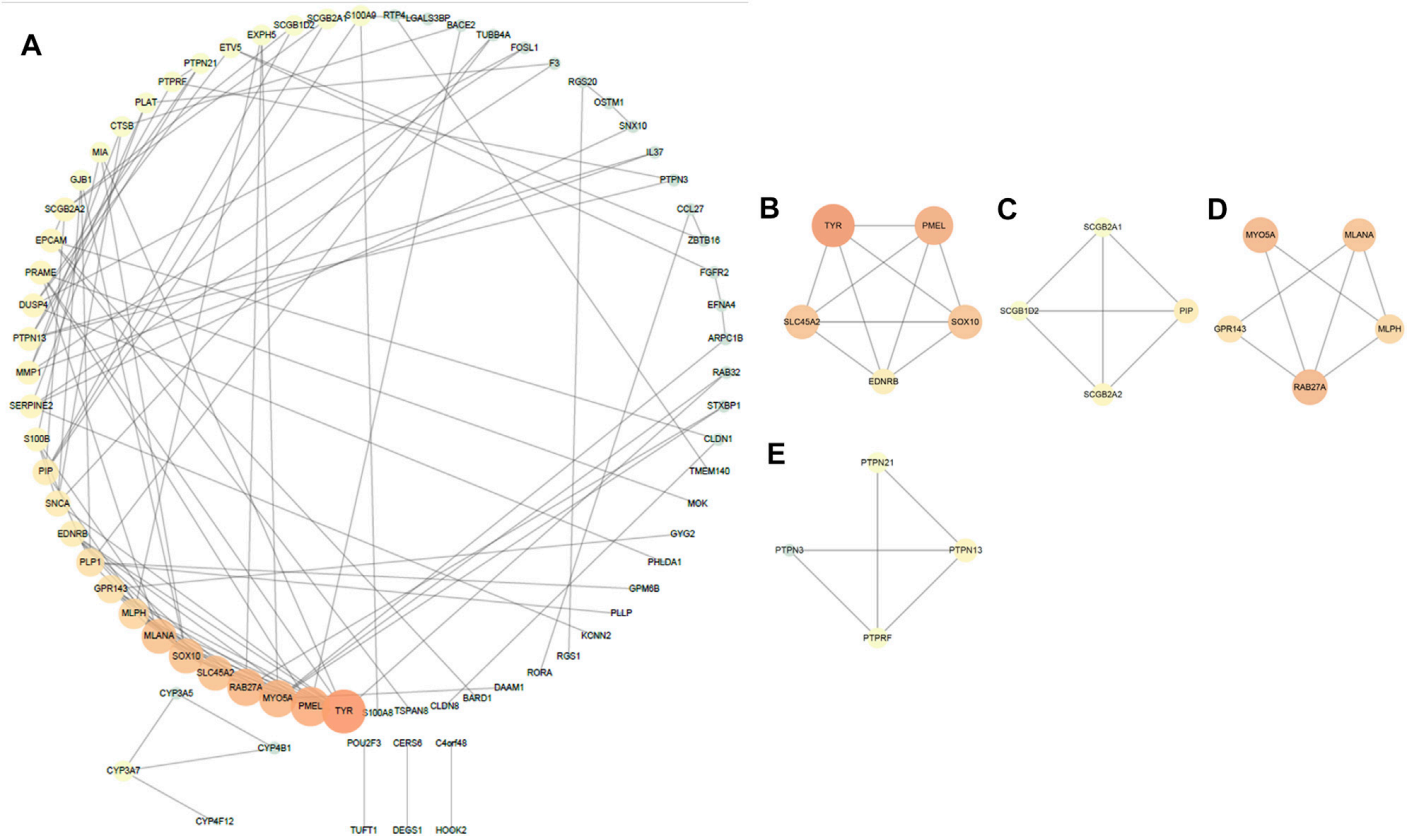
Protein–protein interaction (PPI) network establishment and module analysis of the 135 DEGs. **(A)** PPI network with 75 nodes and 118 edges. **(B–E)** Four modules were identified by MCODE arithmetic.

By analyzing 135 DEGs, we got a network interaction graph with 75 nodes and 118 edges, where nodes represented genes, edges represented connections between two genes, and degree value represented the strength of association between genes. More precisely, the top 10 hub genes of DEGs were TYR, PMEL, RAB27A, MYO5A, MLANA, SOX10, SLC45A2, MLPH, GPR143 and PLP1 (Figure 3A). Four modules were identified by MCODE arithmetic (Figures 3B–E).

### The Validation of Key Genes

**FIGURE 4:**
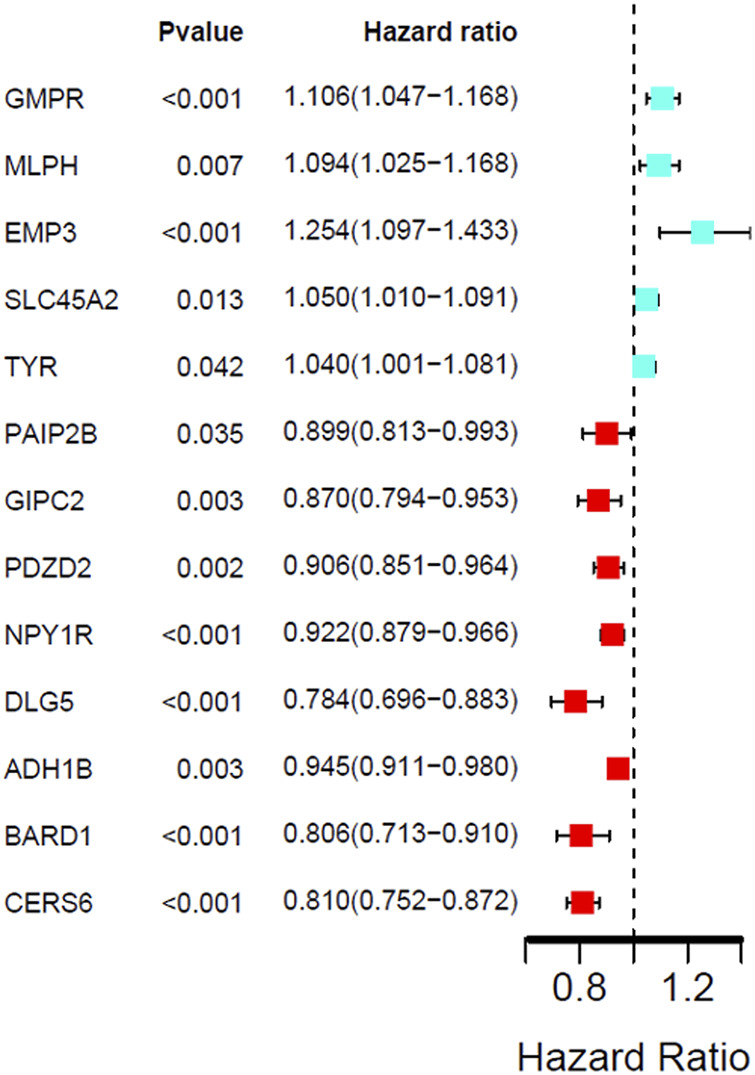
Univariate Cox regression analysis showing the hazard ratios (HRs) with 95% confidence intervals (CIs) and *p* values for 13 DEGs.

**FIGURE 5:**
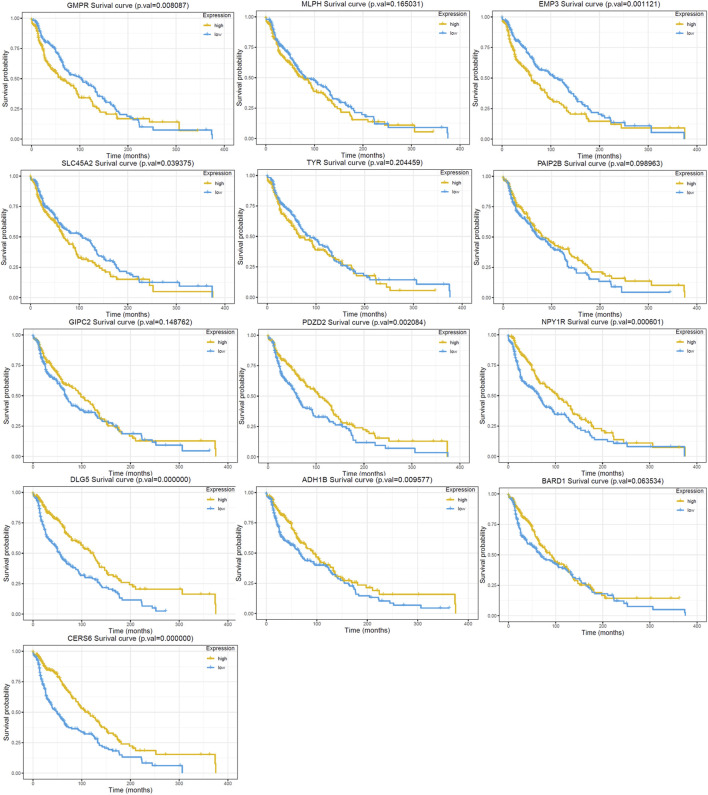
Kaplan Meier survival curve of the 13 DEGs in the RRA analysis. The GMPR, MLPH, EMP3, SLC45A2 and TYR are up-regulated in GEO dataset. The PAIP2B, GIPC2, PDZD2, NPY1R, DLG5, ADH1B, BARD1 and CERS6 are down-regulated in GEO dataset.

**FIGURE 6:**
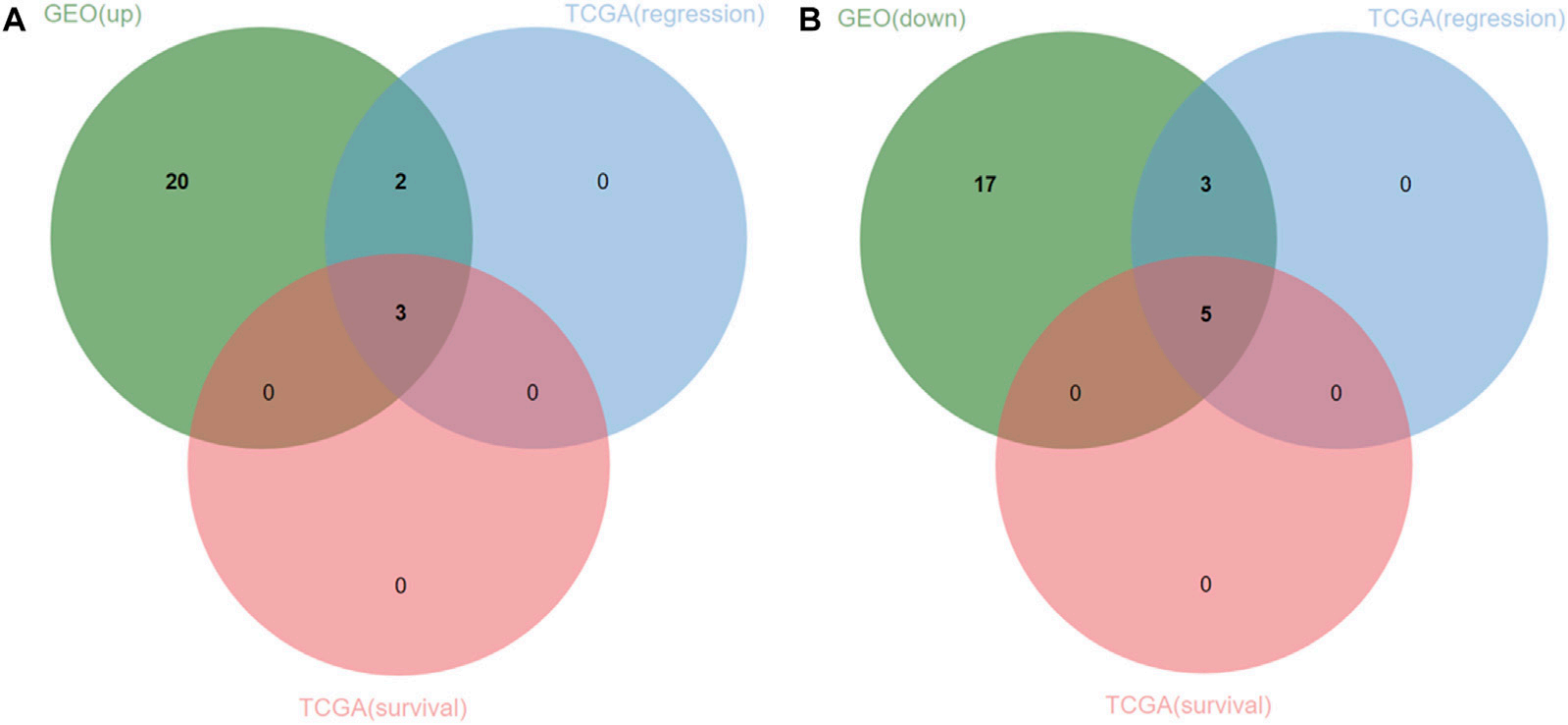
Venn diagram of 25 upregulated and downregulated DEGs and TCGA datasets. **(A)** three upregulated genes (oncogenes) were identified. **(B)** five downregulated genes (tumor suppressor genes) were identified.

The univariate cox regression analysis was used to calculate the Hazard Ratio (HR) of the top 25 up and down-regulated genes for CM patients. The results showed that among these top genes, the expression levels of GMPR, MLPH, EMP3, SLC45A2 TYR, PAIP2B, GIPC2, PDZD2, NPY1R, DLG5, ADH1B, BARD1 and CERS6 was closely related to the survival time of CM patients, with statistically significant differences (*p* < 0.05). HR values of less than one can be translated as PAIP2B, GIPC2, PDZD2, NPY1R, DLG5, ADH1B, BARD1 and CERS6 representing low-risk factors, while GMPR, MLPH, EMP3, SLC45A2 and TYR were high-risk factors (Figure 4). The genes screened by univariate cox regression analysis were used to draw survival curves from Kaplan-Meier estimations, and 8 genes (GMPR, EMP3, SLC45A2, PDZD2, NPY1R, DLG5, ADH1B, CERS6) finally met the requirements according to statistically significant differences (*p* < 0.05). (Figures 5, 6). (Supplementary Table S1).

**FIGURE 7:**
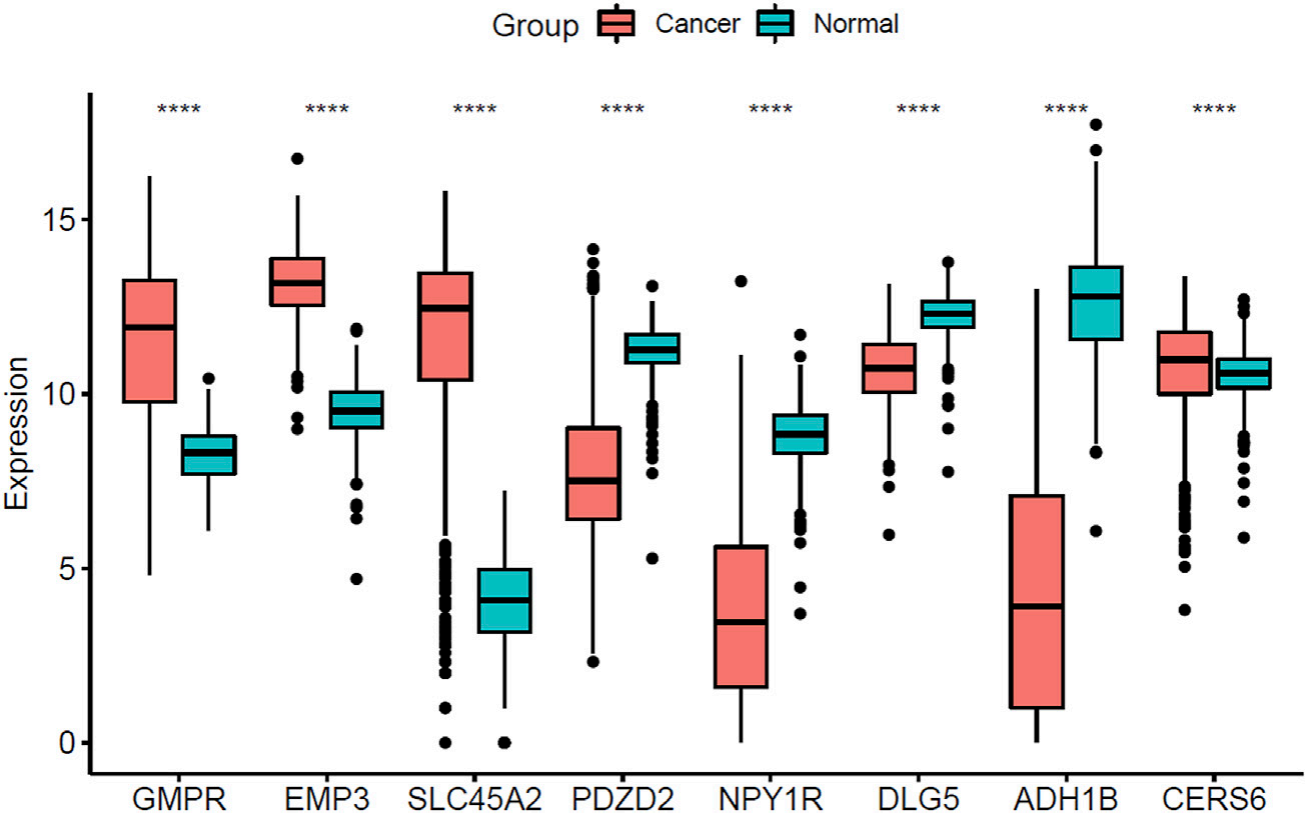
Boxplot of the three screened up-regulated genes (GMPR, EMP3, SLC45A2) and five down-regulated genes (PDZD2, NPY1R, DLG5, ADH1B, CERS6) in TCGA-GTEx dataset. (^****^ *p* < 0.0001).

The expression of 8 hub genes associated with survival time in CM and normal tissues was analyzed in the TCGA- GTEx gene expression dataset and the validation results showed that a total of seven genes showed consistent expression trends in TCGA and GEO datasets, except for CERS6 (CERS6 was up-regulated in CM samples of TCGA dataset and down-regulated in CM samples of GEO dataset) (Figure 7).

**FIGURE 8:**
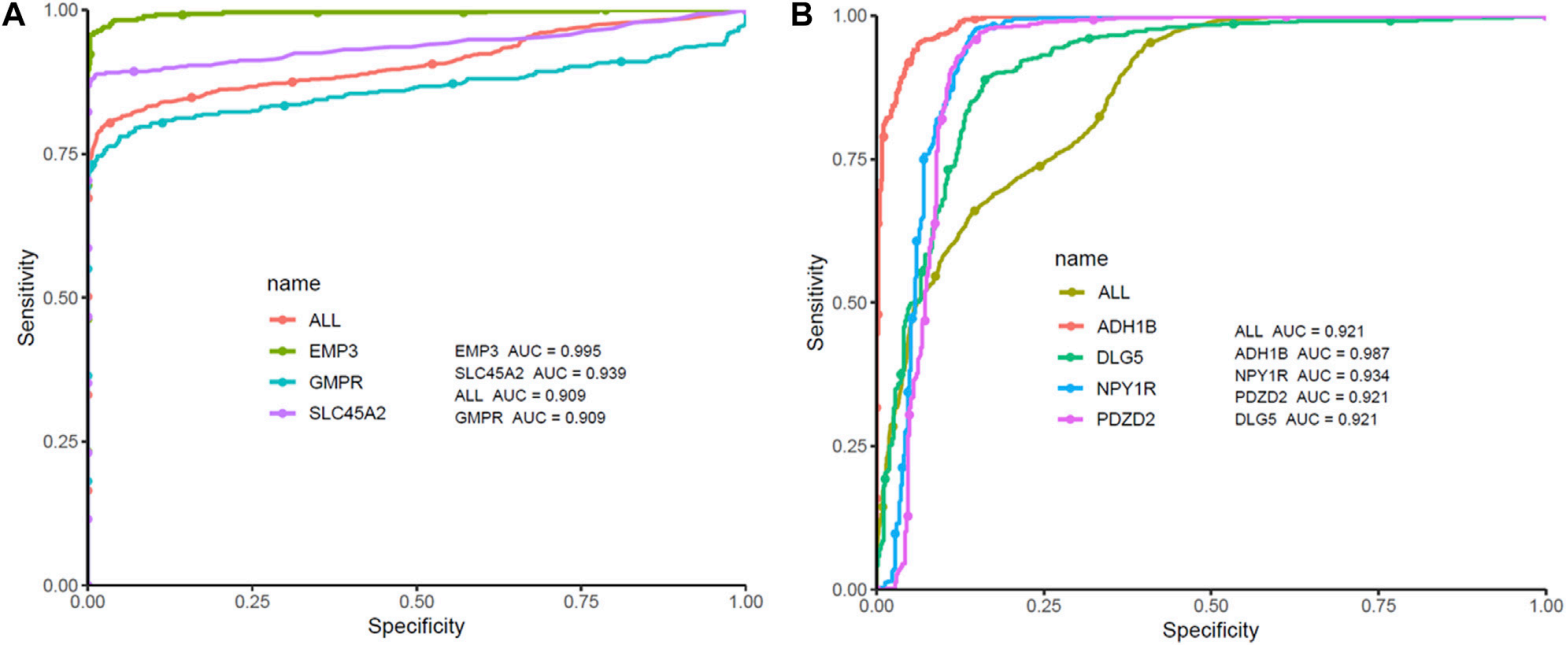
ROC curve analysis and AUC analysis were implemented to evaluate the capacity of seven genes to distinguish CM tissue from normal tissue in TCGA-GTEx dataset. **(A)** ROC curves analysis of three screened up-regulated genes (GMPR, EMP3, SLC45A2). **(B)** ROC curves analysis of four screened down-regulated genes (PDZD2, NPY1R, DLG5, ADH1B).

**FIGURE 9:**
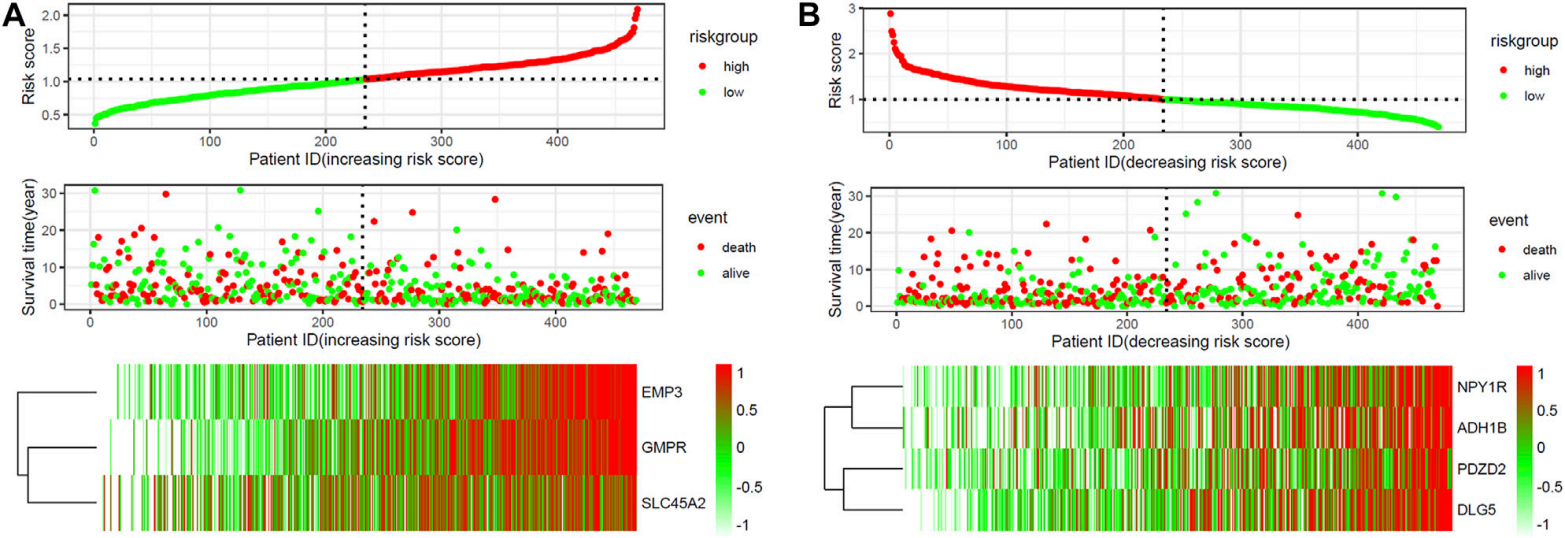
The risk scores for all patients in TCGA cohort are plotted and marked as low risk (blue) or high risk (red), as divided by the threshold (vertical black line). Expression profiles of the DEGs of patients in TCGA cohort, with red indicating higher expression and light blue indicating lower expression. **(A)** three screened up-regulated genes (GMPR, EMP3, SLC45A2). **(B)** four down-regulated genes (PDZD2, NPY1R, DLG5, ADH1B).

ROC curve analysis was performed on seven Hub Genes using the package pROC. AUC >0.9 was taken as the cutoff value, and it was found that the AUC values of these seven genes were all greater than 0.9. The expression levels of these genes have high accuracy in distinguishing normal skin tissue from CM tissue, and could be regarded as potential “tumor biomarkers” for diagnosing CM (Figure 8). Moreover, the distribution of risk scores, survival status, and expression levels of three oncogenes and four tumor suppressor genes are shown in Figure 9.

### Small Molecule Drugs Screening

**FIGURE 10:**
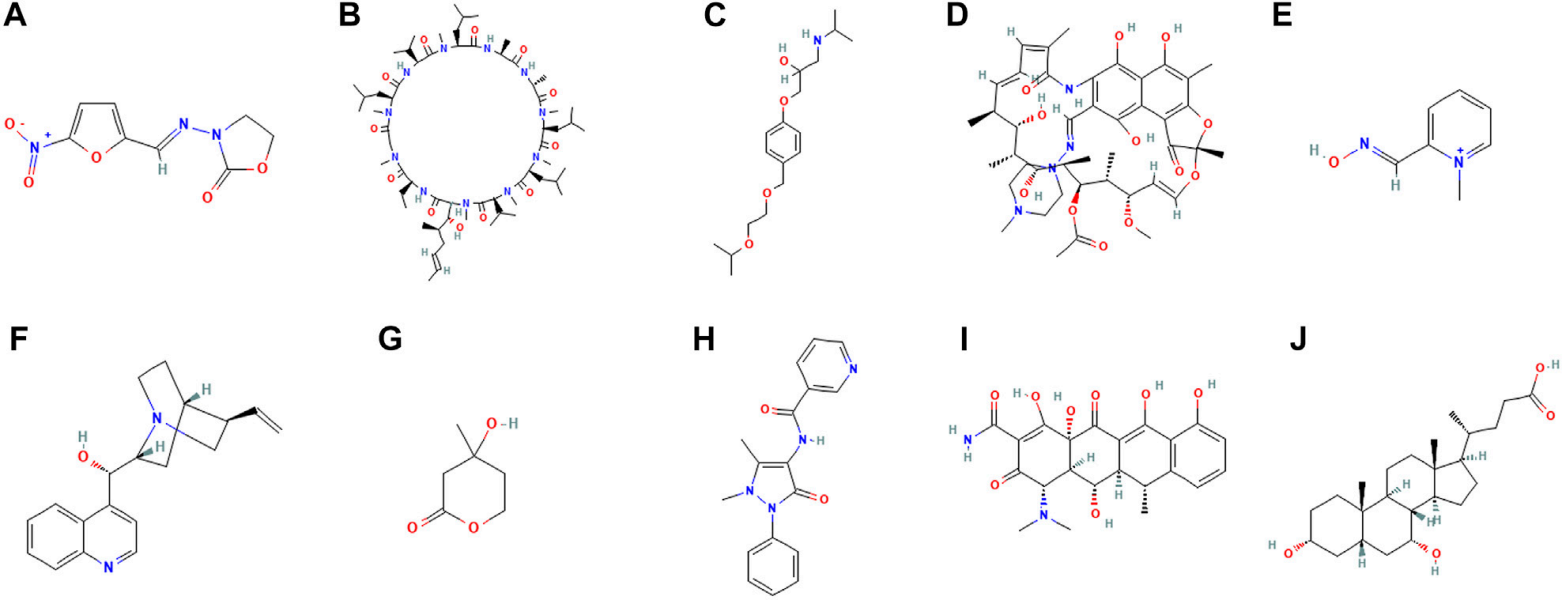
Chemical structure depiction of the top ten most significant drugs. **(A)** Furazolidone **(B)** Ciclosporin **(C)** Bisoprolol **(D)** Rifampicin **(E)** Pralidoxime **(F)** Cinchonine **(G)** Mevalolactone **(H)** Nifenazone **(I)** doxycycline **(J)** chenodeoxycholic acid.

CMap network was used to analyze 7 DEGs into two groups (3 in up-regulated group and four in downregulated group) (Supplementary Table S1). After the signature query, the 10 compounds with the highest negative enrichment score (furazolidone, ciclosporin, bisoprolol, rifampicin, pralidoxime, cinchonine, mevalolactone, nifenazone, doxycycline and chenodeoxycholic acid) were identified as potential therapeutic agents for CM (Table 1). The chemical structures of these ten compounds are shown in Figure 10.

**TABLE 1:** Results of Cmap analysis.

Rank	Cmap name	Mean	N	Enrichment	*p*-value
1	Furazolidone	−0.614	4	−0.841	0.00117
2	ciclosporin	−0.418	6	−0.673	0.00332
3	bisoprolol	−0.294	4	−0.784	0.00438
4	rifampicin	−0.294	4	−0.759	0.00688
5	pralidoxime	−0.403	4	−0.757	0.00712
6	cinchonine	−0.241	4	−0.75	0.00778
7	mevalolactone	−0.543	3	−0.836	0.00871
8	nifenazone	−0.488	5	−0.659	0.01103
9	doxycycline	−0.356	5	−0.646	0.01376
10	chenodeoxycholic acid	−0.545	4	−0.712	0.01387

### Molecular Docking Verification

Using AutoDock Vina 1.1.2 software, the screened small molecule drugs were docked with six core targets (GMPR, EMP3, SLC45A2, NPY1R, DLG5, ADH1B). The crystal structure of PDZD2 was not obtained, so it could not be docked. A binding energy less than 0 indicates spontaneous binding of the ligand and receptor. The lower the binding energy, the more stable the binding conformation and the greater the likelihood of action (Gao et al., 2016).

**FIGURE 11:**
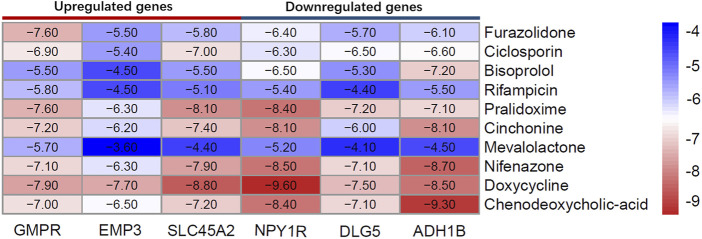
Heat map of the lowest binding energy for molecular docking.

**FIGURE 12:**
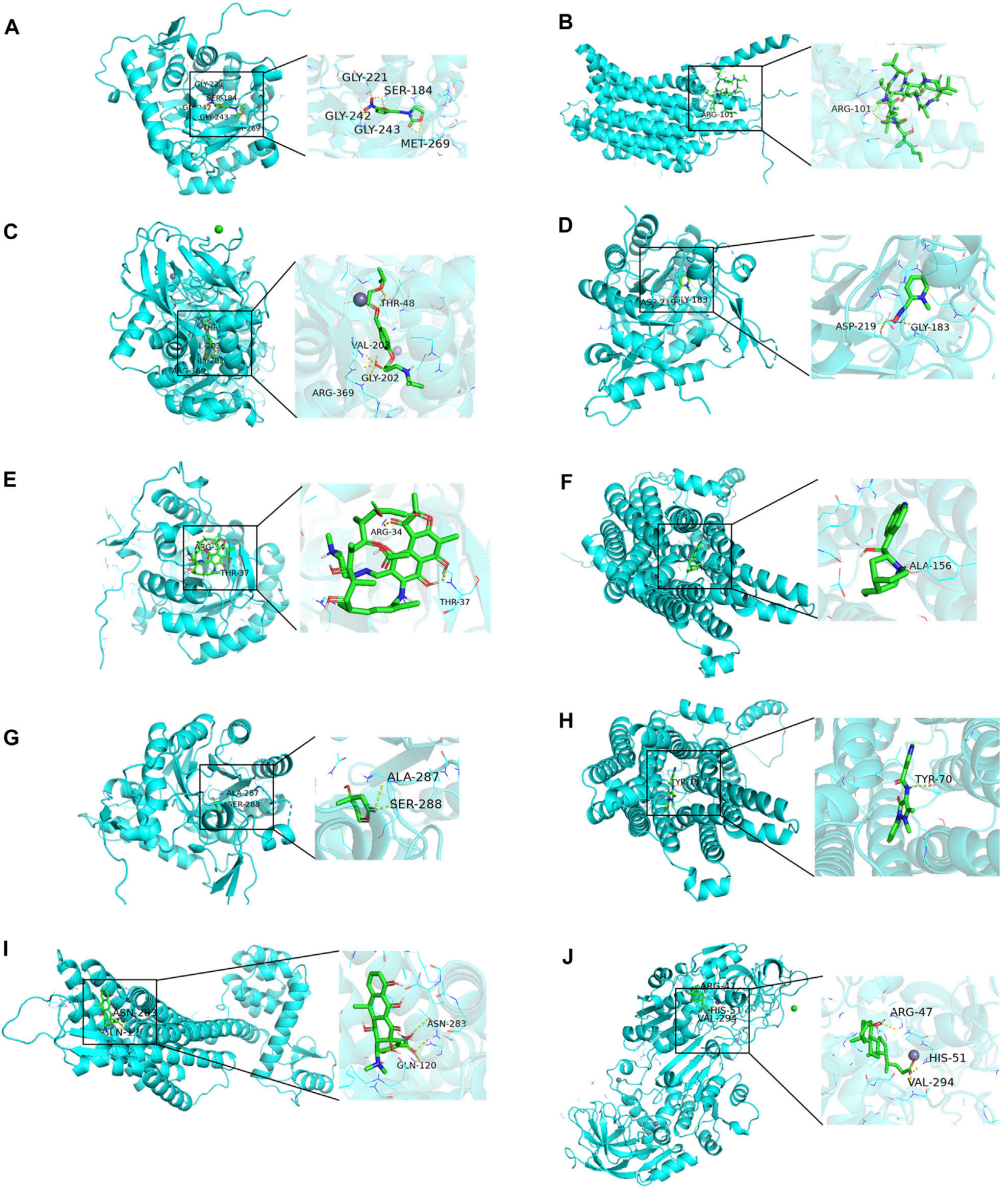
Docking diagram of small molecule drugs with targets. **(A)** Furazolidone-GMPR **(B)** Ciclosporin-SLC45A2 **(C)** bisoprolol-ADH1B **(D)** rifampicin-GMPR **(E)** pralidoxime-NPY1R **(F)** cinchonine-NPY1R **(G)** mevalolactone-GMPR **(H)** nifenazone-SLC45A2 **(I)** doxycycline-NPY1R **(J)** chenodeoxycholic acid-ADH1B.

As can be seen from Figure 11, the minimum binding energy between the ligand and the receptor is mostly less than −7.0 kcal·mol-1, indicating that the target protein has a good affinity with the active ingredient, and small molecule drugs are likely to act on these targets. Small molecule drug docking targets with the lowest binding energy were selected for docking visualization (Figure 12). The dotted lines in the figure are hydrogen bonds. For example, furazolidone exerts its biological efficacy most likely by binding to GMPR and forming hydrogen bonds with the five amino acids GLY221, SER183, GLY242, GLY243 and MET269 near the active site.

## Discussion

CM is a tumor formed by malignant transformation of skin melanocytes. It has the characteristics of high degree of malignancy, strong invasiveness, and can affect all ages. If not actively treated, it is easy to spread and metastasize through the dermis. Therefore, CM patients have poor prognosis and high mortality (Böhme and Bosserhoff, 2016). Although a series of treatment methods such as radiotherapy, chemotherapy, immunotherapy, and targeted therapy have been used to improve the survival rate of patients, problems such as drug resistance, low drug sensitivity and poor prognosis have to be paid attention to (Domingues et al., 2018; Lebbé et al., 2019; Pelster and Amaria, 2019). Therefore, it is urgent to identify more therapeutic targets, prognostic biomarkers, and potential drugs that can treat CM.

We performed an integrated analysis of the 3 CM DEGs using the RRA method. A total of 135 differential genes were identified, including 65 downregulated and 70 upregulated. GO analysis indicated that the DEGs were associated with melanin biosynthetic process, melanosome and RAGE receptor binding. KEGG analysis showed that these DEGs were primarily enriched in Pathways in cancer. These results are consistent with the existing research results of CM, reflecting the close correlation between the DEGs and CM.

PPI analysis shows that Tyrosinase (TYR) is the most associated and core gene in DEGs. TYR is a copper-containing oxidase that regulates melanin synthesis (Chang, 2009). Appropriate expression of tyrosinase is beneficial to human beings, as it is a key biochemical catalyst for biosynthesis of natural melanin to protect skin from photocarcinogenesis. Overexpression of tyrosinase can induce melanoma, and TYR can mediate melanoma cell apoptosis under the regulation of transcription factor MITF (Steingrímsson et al., 2004; Shirasugi et al., 2010; Lee et al., 2015; Liu et al., 2020).

Seven genes (GMPR, EMP3, SLC45A2, NPY1R, DLG5, PDZD2 and ADH1B) were finally screened out by combined analysis of GEO and TCGA databases. Cox regression analysis showed that these genes were independent prognostic indicators of CM patients. Among them, only PDZD2, NPY1R and ADH1B have not been reported in melanoma studies, and EMP3 has only been reported in uveal melanoma (Kaochar et al., 2018).

PDZD2 (PDZ domain containing 2) is a multi-PDZ protein expressed in many tissues (Yeung et al., 2003). We found that PDZD2 was poorly expressed in cutaneous melanoma. Previous studies have suggested that PDZD2 is an oncogene, which is over-expressed in the cell lines of osteosarcoma, human primary prostate tumor and prostate tumor (He et al., 2019). Recently, it has been shown that human secreted PDZD2 (sPDZD2) has anti-tumor properties, and it is also down-regulated in lung adenocarcinoma (Cui et al., 2020). Spdzd2 induces the aging of prostate cancer cells through mutation or transcriptional activation of wild-type p53, and makes cancer cells more sensitive to apoptosis through genotoxic stress (Tam et al., 2006). SPDZD2 plays an antiproliferative role in human cancer cells by affecting cell cycle arrest in S phase (Tam et al., 2008). The difference of PDZD2 expression in different tumor tissues may be related to tissue specificity.

NPY is an important regulator of tumor progression of nerve or endocrine related cancers. The role of NPY may be mediated by many NPY receptor subtypes. Many evidences show that the expression of NPY1R gene is up-regulated in various types of nerve or endocrine related cancers (including breast cancer, prostate cancer, adrenal tumor, renal cell cancer and ovarian cancer, etc.), (Kitlinska et al., 2005; Ruscica et al., 2006; Körner et al., 2008; Lv et al., 2016). Activation of Y1R and Y2R by NPY leads to tumor cell proliferation, angiogenesis and metastasis (Liu et al., 2015). In non-neuroendocrine related tumors such as in hepatocellular carcinoma cells, npy1r inhibit cell proliferation by activating mitogen-activated protein kinase signal pathway, promoting tumor growth and increasing tumorigenicity of cells (Lv et al., 2016).

ADH1B, a member of the alcohol dehydrogenase (ADH) family, is involved in the metabolism of acetaldehyde, a carcinogen (Seitz and Stickel, 2010; Galinsky et al., 2016). ADH1B (no. 1) has been inhibited in almost all cancer types, and the ability of ADH1B to inhibit cancer cells has been confirmed *in vitro* experiments (Li et al., 2017). ADH1B is involved in the metabolism of several anti-tumor drugs, including ifosfamide and cyclophosphamide (Polimanti et al., 2016). Differential expression of ADH1B in ovarian cancer can make cells secrete MMP-7 CD-26 and cathepsin to promote cancer progression (Gharpure et al., 2018).

New use of old drugs has become an important strategy for the development of anti-tumor drugs, which has the advantages of saving development time, cost and improving drug safety. According to CMap database analysis, furazolidone (ranked first), ciclosporin and bisoprolol can be used to treat CM.

Furazolidone (FZD) is a kind of synthetic nitrofuran derivative, which can sterilize or inhibit gram-positive and gram-negative bacteria (Karamanakos, 2013). FZD has also been shown to have antitumor activity in various other cancers (Jiang et al., 2013). FZD has significant antitumor activity against acute myeloid leukemia (AML) (Enzenauer et al., 1990). FZD promotes the apoptosis of AML cells and induces the differentiation of myeloid cells by stabilizing the tumor suppressor protein p53 (Tang et al., 2006). Another paper also showed that FZD has inhibitory activity on hepatoma cells. FZD induces oxidative DNA damage by increasing reactive oxygen species (ROS), thereby inducing HCC cell cycle arrest. Targeting nuclear factor -κB (NFκB) signaling pathway may serve as a predictor of immunotherapy response in melanoma patients (Poźniak et al., 2019). FZD can inhibit NF-κB signaling pathway and induce apoptosis of small cell lung cancer cells (Yu et al., 2020). NF-κB is a melanoma pathogenic factor (Pozniak et al.), which can regulate the transcription of genes involved in cell survival, and inhibition of NF-κB activation has been considered as a strategy for the treatment of melanoma. Therefore, FDZ may also treat melanoma by inhibiting the activation of NF-κB.

At the same time, through molecular connection, it was found that FZD was most likely to treat CM by acting on guanosine monophosphate reductase (GMPR). In this study, GMPR is considered to be an oncogene, and some studies have found that GMPR is a new melanoma invasion inhibitor. This seemingly contradictory conclusion happened simultaneously in MITF characterized as both a melanoma oncogene (McGill et al., 2002; Wellbrock et al., 2008) and an invasion suppressor (Carreira et al., 2006; Arozarena et al., 2011; Cheli et al., 2011; Javelaud et al., 2011; Cheli et al., 2012), which may be a kind of “Rheostat model” (Wawrzyniak et al., 2013). GMPR is the downstream target of MITF (Bianchi-Smiraglia et al., 2017). MITF can promote proliferation at low and medium levels and differentiation at high levels, and such dynamic changes are related to the dynamic changes of tumor microenvironment after tumor cell metastasis and epigenetic changes related to melanoma metastasis caused by tumor microenvironment changes after tumor cell metastasis (Carreira et al., 2006). Therefore, FZD may achieve its therapeutic purpose by acting on GMPR to inhibit CM cell proliferation rather than invasion.

In summary, RRA method was used in this study to systematically analyze three groups of CM gene chip data, and then the TCGA data set was combined to verify and screen, and finally the key DEGs, such as GMPR, EMP3, SLC45A2, NPY1R, DLG5, PDZD2 and ADH1B, were screened out. In this study, furazolidone was predicted and validated as a potential small-molecule drug for the treatment of CM, providing reference for the selection of CM markers, therapeutic targets and therapeutic drugs. In future studies, we will need to verify the target genes and small molecule drugs we have found through experiments.

## Data Availability

The original contributions presented in the study are included in the article/Supplementary Material, further inquiries can be directed to the corresponding authors.

## References

[B1] Arozarena I. Bischof H. Gilby D. Belloni B. Dummer R. Wellbrock C. (2011). In Melanoma, Beta-Catenin Is a Suppressor of Invasion. Oncogene 30, 4531–4543. 10.1038/onc.2011.162 21577209 PMC3160497

[B2] Bader G. D. Hogue C. W. (2003). An Automated Method for Finding Molecular Complexes in Large Protein Interaction Networks. BMC Bioinformatics 4, 2. 10.1186/1471-2105-4-2 12525261 PMC149346

[B3] Bianchi-Smiraglia A. Bagati A. Fink E. E. Moparthy S. Wawrzyniak J. A. Marvin E. K. (2017). Microphthalmia-associated Transcription Factor Suppresses Invasion by Reducing Intracellular GTP Pools. Oncogene 36, 84–96. 10.1038/onc.2016.178 27181209 PMC5112150

[B4] Böhme I. Bosserhoff A. K. (2016). Acidic Tumor Microenvironment in Human Melanoma. Pigment Cel Melanoma Res. 29, 508–523. 10.1111/pcmr.12495 27233233

[B5] Carreira S. Goodall J. Denat L. Rodriguez M. Nuciforo P. Hoek K. S. (2006). Mitf Regulation of Dia1 Controls Melanoma Proliferation and Invasiveness. Genes Dev. 20, 3426–3439. 10.1101/gad.406406 17182868 PMC1698449

[B6] Chang T.-S. (2009). An Updated Review of Tyrosinase Inhibitors. Int. J. Mol. Sci. 10, 2440–2475. 10.3390/ijms10062440 19582213 PMC2705500

[B7] Cheli Y. Guiliano S. Botton T. Rocchi S. Hofman V. Hofman P. (2011). Mitf Is the Key Molecular Switch between Mouse or Human Melanoma Initiating Cells and Their Differentiated Progeny. Oncogene 30, 2307–2318. 10.1038/onc.2010.598 21278797

[B8] Cheli Y. Giuliano S. Fenouille N. Allegra M. Hofman V. Hofman P. (2012). Hypoxia and MITF Control Metastatic Behaviour in Mouse and Human Melanoma Cells. Oncogene 31, 2461–2470. 10.1038/onc.2011.425 21996743

[B9] Cui S. Lou S. Guo W. Jian S. Wu Y. Liu X. (2020). Prediction of MiR-21-5p in Promoting the Development of Lung Adenocarcinoma via PDZD2 Regulation. Med. Sci. Monit. 26, e923366. 10.12659/MSM.923366 32535612 PMC7313425

[B10] Domingues B. Lopes J. Soares P. Pópulo H. (2018). Melanoma Treatment in Review. Immunotargets Ther. 7, 35–49. 10.2147/ITT.S134842 29922629 PMC5995433

[B11] Enzenauer R. J. Stock J. G. Enzenauer R. W. Pope J. West S. G. (1990). Retinal Vasculopathy Associated with Systemic Light Chain Deposition Disease. Retina 10, 115–118. 10.1097/00006982-199004000-00005 2119514

[B12] Galinsky K. J. Bhatia G. Loh P.-R. Georgiev S. Mukherjee S. Patterson N. J. (2016). Fast Principal-Component Analysis Reveals Convergent Evolution of ADH1B in Europe and East Asia. Am. J. Hum. Genet. 98, 456–472. 10.1016/j.ajhg.2015.12.022 26924531 PMC4827102

[B13] Gao J. Liang L. Zhu Y. Qiu S. Wang T. Zhang L. (2016). Ligand and Structure-Based Approaches for the Identification of Peptide Deformylase Inhibitors as Antibacterial Drugs. Int. J. Mol. Sci. 17, 1141. 10.3390/ijms17071141 PMC4964514 27428963

[B14] Gaudreault F. Morency L.-P. Najmanovich R. J. (2015). NRGsuite: a PyMOL Plugin to Perform Docking Simulations in Real Time Using FlexAID. Bioinformatics 31, btv458–3858. 10.1093/bioinformatics/btv458 PMC4653388 26249810

[B15] Gharpure K. M. Lara O. D. Wen Y. Pradeep S. LaFargue C. Ivan C. (2018). ADH1B Promotes Mesothelial Clearance and Ovarian Cancer Infiltration. Oncotarget 9, 25115–25126. 10.18632/oncotarget.25344 29861857 PMC5982754

[B16] He F. Fang L. Yin Q. (2019). miR-363 Acts as a Tumor Suppressor in Osteosarcoma Cells by Inhibiting PDZD2. Oncol. Rep. 41, 2729–2738. 10.3892/or.2019.7078 30896877 PMC6448123

[B17] Javelaud D. Alexaki V.-I. Pierrat M.-J. Hoek K. S. Dennler S. Van Kempen L. (2011). GLI2 and M-MITF Transcription Factors Control Exclusive Gene Expression Programs and Inversely Regulate Invasion in Human Melanoma Cells. Pigment Cel Melanoma Res. 24, 932–943. 10.1111/j.1755-148X.2011.00893.x 21801332

[B18] Jia X. Zhai T. (2019). Integrated Analysis of Multiple Microarray Studies to Identify Novel Gene Signatures in Non-alcoholic Fatty Liver Disease. Front. Endocrinol. 10, 599. 10.3389/fendo.2019.00599 PMC6736562 31551930

[B19] Jiang X. Sun L. Qiu J. J. Sun X. Li S. Wang X. (2013). A Novel Application of Furazolidone: Anti-leukemic Activity in Acute Myeloid Leukemia. PLoS One 8, e72335. 10.1371/journal.pone.0072335 23951311 PMC3739762

[B20] Kaochar S. Dong J. Torres M. Rajapakshe K. Nikolos F. Davis C. M. (2018). ICG-001 Exerts Potent Anticancer Activity against Uveal Melanoma Cells. Invest. Ophthalmol. Vis. Sci. 59, 132–143. 10.1167/iovs.17-22454 29332125 PMC5769500

[B21] Karamanakos P. N. (2013). Possible Role for Furazolidone in the Treatment of Glioblastoma Multiforme. J. BUON 18, 1097. 24344045

[B22] Kitlinska J. Abe K. Kuo L. Pons J. Yu M. Li L. (2005). Differential Effects of Neuropeptide Y on the Growth and Vascularization of Neural Crest-Derived Tumors. Cancer Res. 65, 1719–1728. 10.1158/0008-5472.CAN-04-2192 15753367

[B23] Körner M. Waser B. Reubi J. C. (2008). High Expression of Neuropeptide Y1 Receptors in ewing Sarcoma Tumors. Clin. Cancer Res. 14, 5043–5049. 10.1158/1078-0432.CCR-07-4551 18698022

[B24] Kolde R. Laur S. Adler P. Vilo J. (2012). Robust Rank Aggregation for Gene List Integration and Meta-Analysis. Bioinformatics (Oxford, England) 28, 573–580. 10.1093/bioinformatics/btr709 PMC3278763 22247279

[B25] Lamb J. (2007). The Connectivity Map: a New Tool for Biomedical Research. Nat. Rev. Cancer 7, 54–60. 10.1038/nrc2044 17186018

[B26] Lebbé C. Meyer N. Mortier L. Marquez-Rodas I. Robert C. Rutkowski P. (2019). Evaluation of Two Dosing Regimens for Nivolumab in Combination with Ipilimumab in Patients with Advanced Melanoma: Results from the Phase IIIb/IV CheckMate 511 Trial. J. Clin. Oncol. 37, 867–875. 10.1200/JCO.18.01998 30811280 PMC6455714

[B27] Lee H. Lee W. Chang S. Lee G.-Y. (2015). Hesperidin, A Popular Antioxidant Inhibits Melanogenesis via Erk1/2 Mediated MITF Degradation. Int. J. Mol. Sci. 16, 18384–18395. 10.3390/ijms160818384 26262610 PMC4581251

[B28] Li Q.-G. He Y.-H. Wu H. Yang C.-P. Pu S.-Y. Fan S.-Q. (2017). A Normalization-free and Nonparametric Method Sharpens Large-Scale Transcriptome Analysis and Reveals Common Gene Alteration Patterns in Cancers. Theranostics 7, 2888–2899. 10.7150/thno.19425 28824723 PMC5562223

[B29] Liu L. Xu Q. Cheng L. Ma C. Xiao L. Xu D. (2015). NPY1R Is a Novel Peripheral Blood Marker Predictive of Metastasis and Prognosis in Breast Cancer Patients. Oncol. Lett. 9, 891–896. 10.3892/ol.2014.2721 25624911 PMC4301529

[B30] Liu X. Li H. Cong X. Huo D. Cong L. Wu G. (2020). α-MSH-PE38KDEL Kills Melanoma Cells via Modulating Erk1/2/MITF/TYR Signaling in an MC1R-dependent Manner. Onco Targets Ther. 13, 12457–12469. 10.2147/OTT.S268554 33299329 PMC7721307

[B31] Liu Y. Cui S. Sun J. Yan X. Han D. (2021). Identification of Potential Biomarkers for Psoriasis by DNA Methylation and Gene Expression Datasets. Front. Genet. 12, 722803. 10.3389/fgene.2021.722803 34512732 PMC8427602

[B32] Lv X. Zhao F. Huo X. Tang W. Hu B. Gong X. (2016). Neuropeptide Y1 Receptor Inhibits Cell Growth through Inactivating Mitogen-Activated Protein Kinase Signal Pathway in Human Hepatocellular Carcinoma. Med. Oncol. 33, 70. 10.1007/s12032-016-0785-1 27262566

[B33] McGill G. G. Horstmann M. Widlund H. R. Du J. Motyckova G. Nishimura E. K. (2002). Bcl2 Regulation by the Melanocyte Master Regulator Mitf Modulates Lineage Survival and Melanoma Cell Viability. Cell 109, 707–718. 10.1016/s0092-8674(02)00762-6 12086670

[B34] Pelster M. S. Amaria R. N. (2019). Combined Targeted Therapy and Immunotherapy in Melanoma: a Review of the Impact on the Tumor Microenvironment and Outcomes of Early Clinical Trials. Ther. Adv. Med. Oncol. 11, 175883591983082. 10.1177/1758835919830826 PMC6384439 30815041

[B35] Polimanti R. Kranzler H. R. Gelernter J. (2016). Phenome-Wide Association Study for Alcohol and Nicotine Risk Alleles in 26394 Women. Neuropsychopharmacol 41, 2688–2696. 10.1038/npp.2016.72 PMC5026736 27187070

[B36] Poźniak J. Nsengimana J. Laye J. P. O’Shea S. J. Diaz J. M. S. Droop A. P. (2019). Genetic and Environmental Determinants of Immune Response to Cutaneous Melanoma. Cancer Res. 79, 2684–2696. 10.1158/0008-5472.CAN-18-2864 30773503 PMC6544535

[B37] Ruscica M. Dozio E. Boghossian S. Bovo G. Martos Riaño V. Motta M. (2006). Activation of the Y1 Receptor by Neuropeptide Y Regulates the Growth of Prostate Cancer Cells. Endocrinology 147, 1466–1473. 10.1210/en.2005-0925 16339211

[B38] Saginala K. Barsouk A. Aluru J. S. Rawla P. Barsouk A. (2021). Epidemiology of Melanoma. Med. Sci. 9, 63. 10.3390/medsci9040063 PMC8544364 34698235

[B40] Seitz H. K. Stickel F. (2010). Acetaldehyde as an Underestimated Risk Factor for Cancer Development: Role of Genetics in Ethanol Metabolism. Genes Nutr. 5, 121–128. 10.1007/s12263-009-0154-1 19847467 PMC2885165

[B41] Shannon P. Markiel A. Ozier O. Baliga N. S. Wang J. T. Ramage D. (2003). Cytoscape: a Software Environment for Integrated Models of Biomolecular Interaction Networks. Genome Res. 13, 2498–2504. 10.1101/gr.1239303 14597658 PMC403769

[B42] Shirasugi I. Kamada M. Matsui T. Sakakibara Y. Liu M.-C. Suiko M. (2010). Sulforaphane Inhibited Melanin Synthesis by Regulating Tyrosinase Gene Expression in B16 Mouse Melanoma Cells. Biosci. Biotechnol. Biochem. 74, 579–582. 10.1271/bbb.90778 20208349

[B43] Steingrímsson E. Copeland N. G. Jenkins N. A. (2004). Melanocytes and the Microphthalmia Transcription Factor Network. Annu. Rev. Genet. 38, 365–411. 10.1146/annurev.genet.38.072902.092717 15568981

[B44] Tam C. W. Cheng A. S. Ma R. Y. M. Yao K.-M. Shiu S. Y. W. (2006). Inhibition of Prostate Cancer Cell Growth by Human Secreted PDZ Domain-Containing Protein 2, a Potential Autocrine Prostate Tumor Suppressor. Endocrinology 147, 5023–5033. 10.1210/en.2006-0207 16873542

[B45] Tam C. W. Liu V. W. S. Leung W. Y. Yao K.-M. Shiu S. Y. W. (2008). The Autocrine Human Secreted PDZ Domain-Containing Protein 2 (sPDZD2) Induces Senescence or Quiescence of Prostate, Breast and Liver Cancer Cells via Transcriptional Activation of P53. Cancer Lett. 271, 64–80. 10.1016/j.canlet.2008.05.047 18639375

[B46] Tang X. Liu D. Shishodia S. Ozburn N. Behrens C. Lee J. J. (2006). Nuclear Factor-κB (Nf-κB) Is Frequently Expressed in Lung Cancer and Preneoplastic Lesions. Cancer 107, 2637–2646. 10.1002/cncr.22315 17078054

[B47] Wawrzyniak J. A. Bianchi-Smiraglia A. Bshara W. Mannava S. Ackroyd J. Bagati A. (2013). A Purine Nucleotide Biosynthesis Enzyme Guanosine Monophosphate Reductase Is a Suppressor of Melanoma Invasion. Cel Rep. 5, 493–507. 10.1016/j.celrep.2013.09.015 PMC3902135 24139804

[B48] Wellbrock C. Rana S. Paterson H. Pickersgill H. Brummelkamp T. Marais R. (2008). Oncogenic BRAF Regulates Melanoma Proliferation through the Lineage Specific Factor MITF. PLoS One 3, e2734. 10.1371/journal.pone.0002734 18628967 PMC2444043

[B49] Yeung M. L. Tam T. S. M. Tsang A. C. C. Yao K. M. (2003). Proteolytic Cleavage of PDZD2 Generates a Secreted Peptide Containing Two PDZ Domains. EMBO Rep. 4, 412–418. 10.1038/sj.embor.embor804 12671685 PMC1319160

[B50] Yu J. G. Ji C. H. Shi M. H. (2020). The Anti‐infection Drug Furazolidone Inhibits NF‐κB Signaling and Induces Cell Apoptosis in Small Cell Lung Cancer. Kaohsiung J. Med. Sci. 36, 998–1003. 10.1002/kjm2.12281 32767507 PMC11896496

